# Introductory Lecture on Obstetrics and the Diseases of Women and Children

**Published:** 1847-03

**Authors:** Gunning S. Bradford


***Introductory Lecture on Obstetrics and the Diseases of Women and Children, at the Session of* 1846–'47, *in the Medical Department of the University of New York,*** by Gunning S. Bradford, M. D.

The importance of this branch of medicine, together with the necessity of a most exact knowledge of its practice, is fully impressed and plainly illustrated by cases, the lecture fully sustains the reputation of the Professor as a teacher and writer upon this branch of medicine.

